# Spatial distribution of congenital syphilis in the state of Bahia, Brazil from 2009 to 2018

**DOI:** 10.3389/fepid.2023.1234580

**Published:** 2023-09-08

**Authors:** Caroline Luz Vital, Renato Barbosa Reis, Jorgana Fernanda de Souza Soares, Diego Lopes Paim Miranda, Mitermayer Galvão Reis

**Affiliations:** ^1^ Pathology and Molecular Biology Laboratory, Gonçalo Moniz Institute, Oswaldo Cruz Foundation, Salvador, Bahia, Brazil; ^2^ Department of Medicine of Bahia of Federal University of Bahia, Salvador, Bahia, Brazil; ^3^ Post-Graduate Program in Regional and Urban Development, Salvador University, Salvador, Brazil; ^4^ National Industrial Learning Service University Center and Integrated Center for Manufacturing and Technology (SENAI CIMATEC), Salvador, Bahia, Brazil; ^5^ Department of Preventive and Social Medicine of Faculty of Medicine, Federal University of Bahia, Salvador, Bahia, Brazil; ^6^ PhD in Public Health from the Post-Graduate Program of Collective Health at Federal University of Bahia, Salvador, Bahia, Brazil; ^7^ Post-Graduate Program in Medicine and Health at the Federal University of Bahia, Salvador, Bahia, Brazil; ^8^ Department of Epidemiology of Microbial Diseases, School of Public Health, Yale University, New Haven, CT, United States

**Keywords:** congenital syphilis, prenatal care, time series studies, geographic mapping, ecoepidemiology

## Abstract

**Objective:**

To describe the temporal trend and spatial distribution of congenital syphilis (CS) cases in the state of Bahia, Brazil between 2009 and 2018.

**Method:**

Mixed ecological study conducted through the analysis of data obtained from the Notifiable Diseases Information System and the Live Birth Information System. Global Moran Index I was performed in order to analyze spatial autocorrelation of CS cases in the municipalities of Bahia and the Local Spatial Association Indicator (LISA) was used to identify the formation of spatial regimes in the GeoDA software.

**Results:**

8,786 cases of CS were registered in the period. An increasing growth in CS incidence, with a 511% increase between 2009 and 2018. Spatial autocorrelation was observed between the municipalities (I Moran = 0.452; *p* < 0.001) and four clusters were identified. More frequently, mothers were aged 20–29 years (50.7%); had incomplete primary education (54.9%); were Black and multiracial (93.2%); received prenatal care (82.2%); 49.0% were diagnosed with syphilis during prenatal care; 68.8% were not adequately treated, and 81.1% of their partners were not treated.

**Conclusion:**

The results showed that CS consolidates as a serious public health problem in Bahia, with an incidence 8.4 times higher in the period than the WHO target of 0.5/1,000 live births, predominantly related to inadequate prenatal care and social vulnerability indicators: young mothers with low education levels, as well as individuals identified as Black and multiracial. Thus, programs aimed at women of childbearing age and pregnant women need to be intensified.

## Introduction

Congenital syphilis is an infectious disease caused by the bacterium Treponema pallidum. In 80% of the cases, it is acquired through vertical transmission via the transplacental route from syphilis-infected pregnant women, or through contact of the newborn with the transmitting agent within the birth canal, considering the presence of maternal syphilitic lesions. Fetal infection in the uterus can occur at any stage of syphilis in pregnant women, but especially when there are longer periods of intrauterine exposure as well as during the early, primary or secondary stages of syphilis (1).

In 40% of cases, syphilis vertical transmission can result in abortion (with a higher risk in the first trimester of pregnancy), neonatal deaths, prematurity, low birth weight and early or late congenital manifestations (1–3). It is worth mentioning that specific and simple tests performed during prenatal care and the administration of penicillin on diagnosed gestational cases show excellent price-performance ratios in preventing CS (1, 4).

Since 1986, Brazil has been making significant efforts measures in order to eliminate CS, since it was included on the list of diseases requiring a mandatory report (5). Furthermore, in 1995 Brazil signed the Plan of Action for Elimination of Congenital Syphilis in Latin America and the Caribbean, with a commitment to its eradication and a target of up to 0.5 cases/1,000 live births (LB), including stillbirths, until 2015 (6, 7). Since this target was not reached, in 2016 the deadline was extended to 2020, which to date has not yet been achieved (8, 9).

In 2016, global incidence of CS was estimated at 4.73/1,000 LB, while in the Americas, incidence was estimated at 3.4/1,000 LB (4), with Brazil being responsible for up to 85% of the cases on the American continent (10). With an increasing growth over the years, in 2018 Brazil presented an incidence of 9 cases/1,000 LB (11), an ocurrence far beyond the Pan American Health Organization (PAHO) and the World Health Organization (WHO) targets (8, 9). In the same year, the state of Bahia occupied the 16th position in the state ranking, concentrating the highest incidence in the country (7.4/1,000 LB) (11).

There is an effort to reduce incidence of CS in several countries led by the WHO. Even though, the opposite has been seen worldwide, especially among low-income countries (4). In order to reduce incidence of CS and list intervention priorities, it is necessary to define its spatial distribution and magnitude. Thus, the present study aims to describe the temporal trend and spatial distribution of CS in the state of Bahia, Brazil, between 2009 and 2018.

## Material and methods

Mixed ecological study whose data sources were the individual notification records of congenital syphilis obtained from the Notification of Diseases Information System (SINAN) and the live birth certificates from the Live Birth Information System (SINASC) between 2009 and 2018 for the state of Bahia. Located in the Northeast region, Bahia is the largest and most populous state in the region, and the fifth largest in the country, with an area of 564,722.611 km², divided into 417 municipalities, and an estimated population of 14,930,634 people in 2020. The data were collected between August 15th and October 25th, 2020.

For surveillance purposes, in Brazil the definition of a CS case follows the conditions established by the Informative Note No. 2-SEI/2017:

*Situation 1*: Every newborn, stillbirth, or miscarriage of a woman with untreated or inadequately treated syphilis. Appropriate treatment: Complete treatment according to clinical stage of syphilis with benzathine penicillin, as long as initiated up to 30 days before delivery. Pregnant women who do not fit these criteria will be considered as inappropriately treated. *Situation 2*: Every child under 13 years of age with at least one of the following: - Clinical, cerebrospinal fluid or radiological manifestation of congenital syphilis AND a positive non-treponemal test; - Non-treponemal test titers of the infant greater than those of the mother, in at least two dilutions of simultaneously collected peripheral blood samples; - Ascending non-treponemal test titers in at least two dilutions; - Positive titers of non-treponemal tests after 6 months of age, except in therapeutic follow-up; - Positive treponemal tests after 18 months of age without a previous diagnosis of congenital syphilis. In this situation, the possibility of acquired syphilis must always be ruled out. *Situation 3*: Microbiological evidence of infection by Treponema pallidum in nasal discharge, skin lesion, biopsy or autopsy of a child, miscarriage or stillbirth. Detection of Treponema pallidum by microscopy (dark-field microscopy or microscopy with stained material) (11).

In order to calculate incidence of CS, the number of cases reported between 2009 and 2018 was divided by the total of LB in the period, determined by place of maternal residence, year-to-year, multiplied by 1,000 LB. The definition of a CS case followed the criteria adopted by Brazilian Ministry of Health (12). However, in this work, as these are aggregated data, which do not allow separation according to age group, 34 cases were considered in individuals over 01-year-old in the variable description.

For spatial analysis, the 417 municipalities in the state of Bahia were used as the units of analysis. Spatialization was performed using information from place of maternal residence and georeferenced to the spatial reference unit, in this case, the representative polygons of each municipality in the state of Bahia. For exploratory analysis of spatial data, the definition of neighborhood degree was adopted as weight matrix, which was based on the criteria of contiguity (13). This matrix can be defined as W*ij* = {1 if *i* and *j* are contiguous; 0 if *i* and *j* are not contiguous}. In this study, several weight matrices were tested with different neighborhood criteria, including different higher orders, in order to identify the one that presented the highest and most significant value of the Global Moran Index I (Formula 1) (13):

Global Moran Index (*I*)

(1) $$I=\frac{n}{So}\frac{\sum i\sum j\;WijZiZj}{\sum {\;}_{i=1}^{n}Zi^{2}}$$

*n* = number of municipalities;

*Z* = value of the standardized variable of interest defined according to the spatial weight matrix, W.

This study implemented the Queen matrix method. In order to perform a further detailed analysis of spatial dependence, the local indicator of spatial association (LISA) (Formula 2) was used, which made it possible to identify *clusters*, *outliers* and the presence of more than one spatial regime (13):

LISA

(2) $$Ii=Zi\sum_{\;j=1}^{j}WijZj$$

*W* = form of contiguity between spatial units.

Nine hundred and ninety-nine random permutations were performed to simulate the value with statistical association with the aim to estimate Global Moran Index I. The spatial regimes with statistically significant associations are highlighted on the map in four groups according to details made by LISA: (a) *high-high*: both the analyzed municipality and its neighbor cities present with high incidence; (b) *low-low*: both the analyzed municipality and its neighbor cities present with low incidence; (c) *low-high*: the analyzed municipality present with low incidence and its neighbors cities present with high incidence; (d) *high-low*: the analyzed municipality present with high incidence and its neighbors cities present with low incidence. A significance level of 5% was adopted for both tests (*α* = 0.05). GEODA software (Version 1.12.1.131) was utilized for statistical analysis and elaboration of maps.

In order to characterize CS cases, maternal sociodemographic variables were described: age group (10–14 years old; 15–19 years old; 20–29 years old; 30–39 years old; and 40 years old or more), race (White; Black; multiracial; Asian American; and Indigenous), education level (illiterate; incomplete elementary school; complete elementary school to incomplete secondary school; complete secondary school; and bachelor degree), prenatal care (yes; no), time of maternal syphilis diagnosis (during prenatal care; during childbirth/curettage; after childbirth; and not performed); maternal syphilis treatment regimen (adequate; inadequate; and not performed); partner treatment (yes; no); and final diagnosis of CS (recent CS; late CS; and abortion or stillbirth associated with CS). Data were presented through absolute and relative frequencies and the temporal trend of CS incidence was verified using line graphs. Graphs and tables were generated using Microsoft Excel 2014.

Mixed ecological studies compare temporal trends of a health event between different geographical locations. It brings considerable advantages, such as its fast development and low execution cost, since it is based mostly on big data for elaboration. This type of study also consolidates high power to survey environmental etiological hypotheses and it can measure socio-environmental effects of the implementation of public health programs or policies, as well as their needs. Considering its disadvantages, it is possible to elucidate the variable quality of data recorded from the information systems and the impossibility of establishing an association between an exposure and an outcome due to aggregated data—there is no access to individuals. Also, there is a possibility that the observations are not independent in time (temporal autocorrelation) or that neighboring locations present very similar health indicators due to similar socio-environmental characteristics (14).

## Results

**Figure 1:**
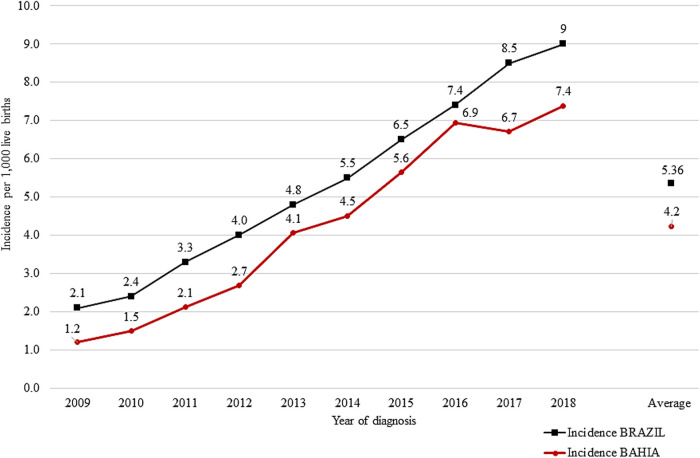
Incidence of congenital syphilis/1,000 LB per year of diagnosis, State of Bahia (red) and Brazil (black), 2009–2018. Sources: SINAN, SINASC (2009–2018). Updated on 30/06/2019.

In the state of Bahia, between 2009 and 2018, 8,786 cases of CS were reported to SINAN, with an estimated incidence of 4.2/1,000 LB, 21.19% under the national incidence. An increasing growth was described during the study period, with an increment of 329% in Brazil and 512% in the state of Bahia, and an emphasis on 2013, when the state demonstrated an increase of 51.9% compared to the previous year. Since 2016, it has been observed a stabilization in the incidence of congenital syphilis in Bahia, with relatively constant rates until 2018. In contrast, Brazil showed continuous growth within the same period. In 2017, Bahia recorded a decrease of 2.9% when compared to 2016, while the country showed an increase of 14.9% compared to the same base year (Figure 1).

**Figure 2:**
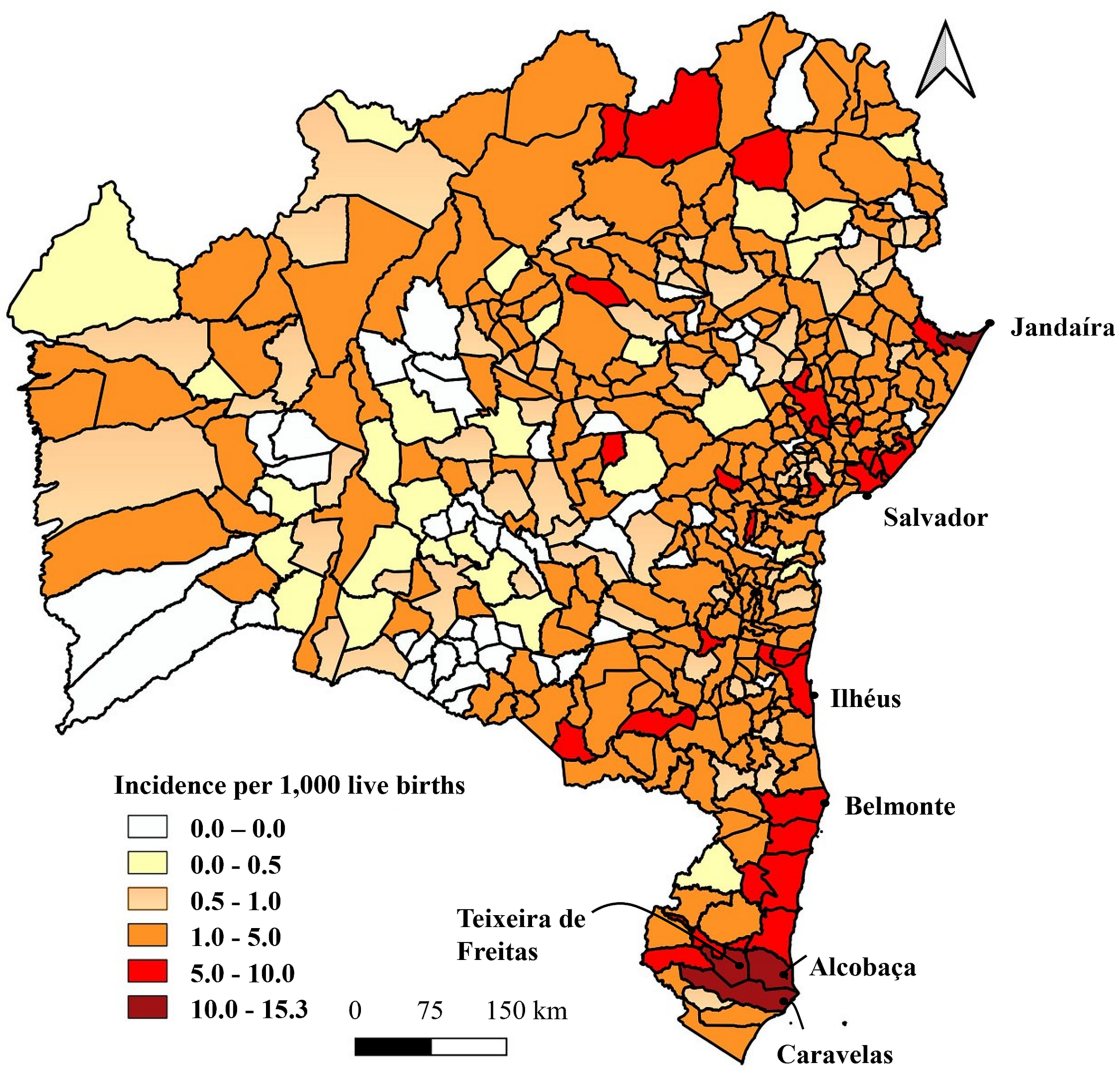
Spatial distribution of the incidence of congenital syphilis/1,000 LB, State of Bahia, 2009–2018. Sources: SINAN, SINASC (2009–2018). Updated on 30/06/2019.

Most municipalities (87.5%; *n* = 365) reported cases of CS. The fifty-two municipalities that did not report any cases of CS are located in the center-west of the state. Four municipalities presented with incidences above 10/1,000 LB: Alcobaça (15.3/1,000 LB); Teixeira de Freitas (12.5/1,000 LB); Jandaira (10.3/1,000 LB); and Caravelas (10.1/1,000 LB), most located in the extreme south of the state. The highest coefficient was presented by Alcobaça, 3.6 times higher compared to the state and 153 times higher than Monte Santo, the city with the lowest coefficient among those with reported cases (0.1/l,000 LB) (Figure 2).

**Figure 3:**
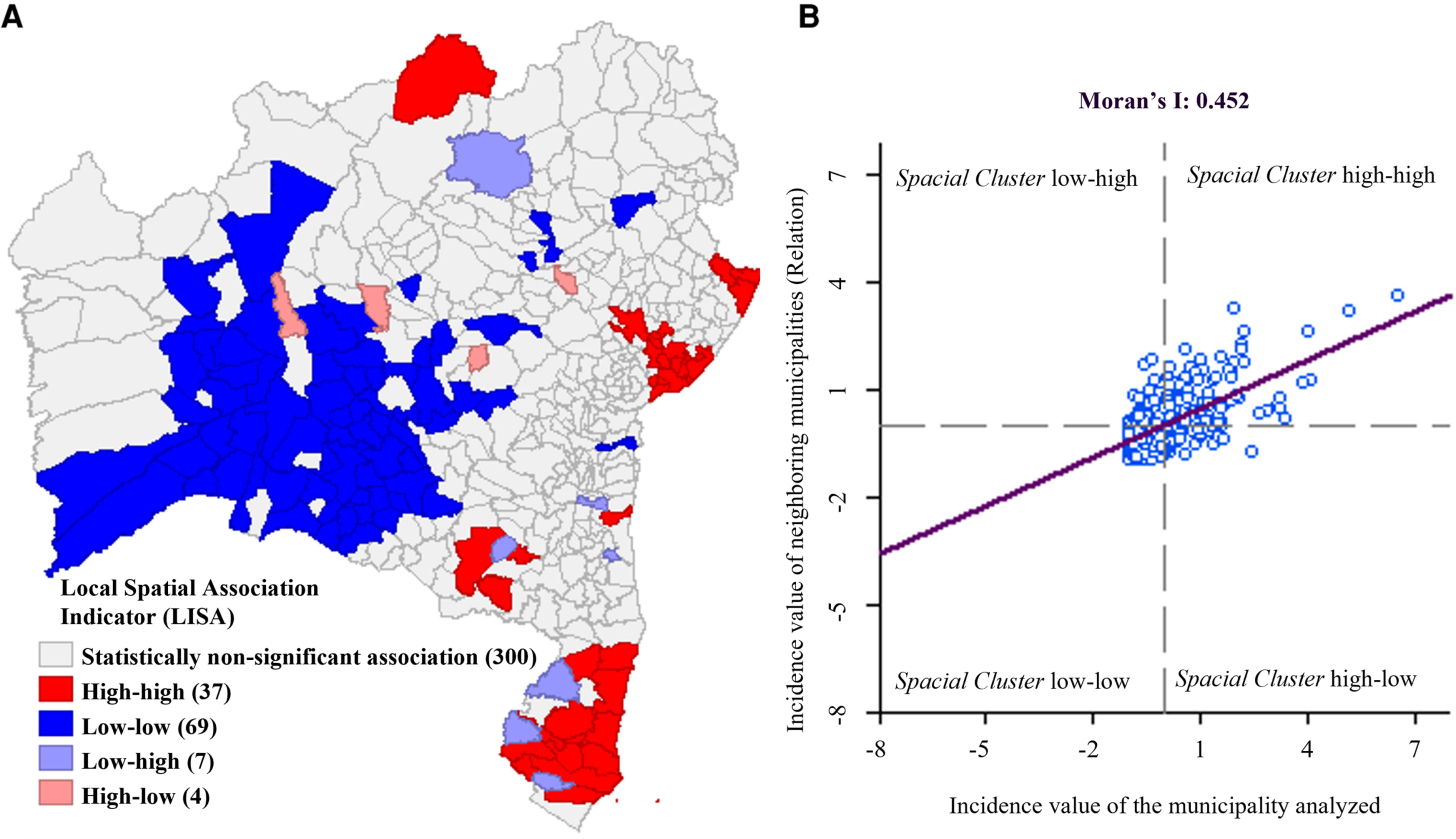
(**A**) Local indicator of spatial association (LISA) of congenital syphilis incidence, State of Bahia, 2009–2018. (**B**) Scatter diagram of Global Moran Index I. Sources: SINAN, SINASC (2009–2018). Updated on 30/06/2019.

Spatial autocorrelation was observed between the municipalities (I Moran = 0.452; *p* < 0.001). Regarding classification of spatial regimes, 37 municipalities were classified as high-high, totaling 5,984 cases, most mentionably Alcobaça, Teixeira de Freitas, Caravelas and Salvador (*p* = 0.001) and Jandaíra (*p* = 0.05). The lowest incidence of the cluster was registered in Casa Nova (2.17/1,000 LB), while the average incidence of this group was 7.46 cases per 1,000 live births. Sixty nine municipalities were classified as low-low, with a total of 138 cases (average incidence of 0.73/1,000 LB) and incidence levels ranging from 0.0/1,000 LB in 23 municipalities (*p* < 0.05) to 1.98/1,000 LB in Ruy Barbosa (*p* = 0.048). Aurelino Leal (1.87/1,000 LB); Barra do Choça (1.41/1,000 LB); Buerarema (1.14/1,000 LB); Campo Formoso (1.68/1,000 LB); Guaratinga (0.36/1,000 LB); Ibirapuã (0.92/1,0000 LB) and Itanhém (1.35/1,000 LB) were considered low-high (39 cases with *p* ≤ 0.047 and average incidence of 1.40/1,000 LB). Barra do Mendes (3.22/1,000 LB); Capela do Alto Alegre (3.82/1,000 LB); Ibiquera (6.99/1,000 LB) and Ibotirama (2.11/1,000 LB) were classified as high-low (23 cases with *p* ≤ 0.045 and average incidence of 2.97/1,000 LB). The incidences of the remaining municipalities did not form clusters (*p* > 0.05) (Figure 3).

Regarding maternal characteristics, age group 20–29 years old was the most common in the state of Bahia (50.7%), following the same profile in the studied groups, however, the high-low cluster also presented a large percentage of maternal age between 15 and 19 years old (40.9%). In terms of maternal education level, incomplete elementary school was the most common in the state of Bahia (54.9%), with the same pattern observed in the clusters. Most mothers were classified as Black and multiracial both in Bahia (93.2%) and among the clusters (Table 1).

**Table 1:** Maternal characteristics according to LISA groups.

Variable	LISA								State of Bahia	
	High-high		Low-low		Low-high		High-low			
	*n*	%	*n*	%	*n*	%	*n*	%	*n*	%
Maternal characteristics										
Age group (years) (*n* = 8.461)										
10–14 years old	47	0.8	0	0	1	2.6	1	4.5	84	1
15–19 years old	1,304	22.8	36	26.3	11	28.9	9	40.9	2,009	23.7
20–29 years old	2,874	50.2	59	43.1	17	44.7	10	45.5	4,289	50.7
30–39 years old	1,355	23.7	40	29.2	7	18.4	2	9.1	1,878	22.2
40 years old or more	145	2.5	2	1.5	2	5.3	0	0	201	2.4
Maternal education level (*n* = 5.505)										
Illiterate	34	0.9	1	1	2	7.1	0	0	75	1.4
Incomplete elementary school	1,966	53	51	53.1	19	67.9	11	64.7	3,024	54.9
Complete elementary school to incomplete secondary school	952	25.7	28	29.2	5	17.9	3	17.6	1,365	24.8
Complete secondary school	684	18.4	16	16.7	2	7.1	3	17.6	947	17.2
Bachelor degree	74	2	0	0	0	0	0	0	94	1.7
Maternal race (*n* = 7.492)										
White	269	5.4	12	9.2	3	8.1	1	4.5	439	5.9
Black	1,089	21.7	26	20	3	8.1	3	13.6	1,382	18.4
Asian American	38	0.8	1	0.8	0	0	0	0	49	0.7
Multiracial	3,617	72	91	70	31	83.8	18	81.8	5,606	74.8
Indigenous	13	0.3	0	0	0	0	0	0	16	0.2

State of Bahia, 2009–2018. Source: SINAN (2009–2018). Updated on 30/06/2019.

In the state of Bahia, most mothers received prenatal care (82.2%) and diagnosis of syphilis was most frequent during prenatal care (49.0%). Both results were also found in the clusters, with evidence for the high-low that achieved 100% coverage in prenatal care and a 91.3% diagnosis at this time. Maternal treatment regimen was found to be inadequate in Bahia (68.8%) and in the clusters. Most partners were not treated in the state of Bahia (81.1%) and among high-high LISA municipalities (83.7%), the same occurring in those classified as low-low, although in a smaller proportion (68.1%). Regarding the final diagnosis of CS, most were classified as recent CS in the state of Bahia (96.3%) as well as among high-high (96.1%) and low-low (98.6%) LISA municipalities (Table 2).

**Table 2:** Prenatal care characteristics according to LISA groups.

Variable	LISA								State of Bahia	
	High-high		Low-low		Low-high		High-low			
	*n*	%	*n*	%	*n*	%	*n*	%	*n*	%
Prenatal care characteristics										
Performance of prenatal (*n* = 7.659)										
Yes	3,973	78.5	129	95.6	31	81.6	23	100	6,296	82.2
No	1,086	21.5	6	4.4	7	18.4	0	0	1,363	17.8
Time of maternal syphilis diagnosis (*n* = 8.156)										
During prenatal care	2,577	47.1	89	65.4	19	51.4	21	91.3	3,994	49
During childbirth/curettage	2,074	37.9	25	18.4	13	35.1	0	0	2,804	34.4
After childbirth	787	14.4	21	15.4	5	13.5	2	8.7	1,306	16
Not performed	34	0.6	1	0.7	0	0	0	0	52	0.6
Maternal syphilis treatment regimen (*n* = 7.335)										
Adequate	171	3.4	13	10.1	3	9.7	2	9.1	300	4.1
Inadequate	3,474	69.2	83	64.3	20	64.5	17	77.3	5,044	68.8
Not performed	1,374	27.4	33	25.6	8	25.8	3	13.6	1,991	27.1
Partner treatment (*n* = 6.258)										
Yes	668	16.3	37	31.9	9	30	10	50	1,185	18.9
No	3,425	83.7	79	68.1	21	70	10	50	5,073	81.1
Final diagnosis of congenital syphilis (*n* = 8.820)										
Recent congenital syphilis	5,770	96.1	137	98.6	38	97.4	22	95.7	8,491	96.3
Late congenital syphilis	13	0.2	1	0.7	0	0	0	0	26	0.3
Abortion associated with congenital syphilis	111	1.8	0	0	0	0	0	0	135	1.5
Stillbirth associated with congenital syphilis	109	1.8	1	0.7	1	2.6	1	4.3	168	1.9

State of Bahia, 2009–2018. Source: SINAN (2009–2018). Updated on 30/06/2019.

## Discussion

The present study demonstrated that CS consolidates as a serious public health problem in the state of Bahia, with 8.4 times higher incidence in the analyzed period than the WHO target of 0.5/1,000 (8, 9). Higher CS rates in the state and among *high-high* clusters may be associated to the precarious prenatal care and mother's social vulnerability indicators, such as young age, low education levels, and identification as Black or multiracial, in accordance with results observed in other studies (15–21). These characteristics can impact on the inequality of access to health services and also on therapeutic adherence. Thus, programs aimed at women of childbearing age and pregnant women need to be intensified in the Primary Health Care, in order to implement effective measures to prevent CS.

The significant increase in the number of CS cases may be associated to several factors, such as the improvement in the notification and surveillance system, the higher coverage of rapid tests, the decreased condom use (22) and the shortage of penicillin in Brazil between 2014 and 2016 (22, 23). Therefore, this epidemic requires prioritization by Public Health managers, given the potential permanent consequences of CS, which require specialized attention throughout the patient's life.

A group of municipalities in the extreme south of the state presented coefficients above the WHO target: 30.6 times higher in Alcobaça; 25 times higher in Teixeira de Freitas; and 20.2 times higher in Caravelas (8, 9). These data reveal aspects beyond health indicators and denote socioeconomic and geographic characteristics of the locations. When considering this area, it is important to highlight the intense daily flow of people through the road junction in the region.

On the other hand, the high incidence described in Salvador (19.7 times higher than the WHO target) also demonstrates important aspects. The state capital presents important cultural events, such as Carnival, which attract many tourists from all over the country and the world, thereby it may be an important factor for the disease transmission. Another issue may be related to the high concentration of health regulation services in the city, which also presents two offices of the Reference Center for Diagnosis, Care and Research (CEDAP), a reference center for the treatment of sexually transmitted infections and an important center for the care of people infected with syphilis (24).

This study demonstrates that spatial distribution is associated with the incidence of syphilis vertical transmission. On the other hand, a study on CS in the state of Goiás (25) that also used the Global Moran Index did not find formation of spatial regimes or spatial autocorrelation for the phenomenon of CS.

Discrepant results were found among the incidences of municipalities in the state of Bahia with reported cases, with 333 cities presenting incidences above the WHO target and only 32 cities presenting incidences as recommended (8, 9). Several factors might be related to the low coefficient, such as patient transfer for other municipalities, erroneous notifications, inadequate data input on SINAN and distribution of specialized health centers. Alternatively, municipalities with higher incidence levels may have greater diagnostic capacity or failures in the prenatal follow-up process.

The reduction of vertical transmission is associated with the correct performance of the serological screening test (*Venereal Disease Research Laboratory—VDRL*) during prenatal care and adequate treatment of pregnant women and partners with benzathine penicillin (17, 19–21, 26). However, failures in the diagnosis and treatment of maternal syphilis and disparities in the quality of care for pregnant women negatively interfere with the reduction of cases. In the present study, it was identified that in the *high-high* cluster, many pregnant women were diagnosed only at the moment of delivery/curettage, and an extremely low proportion of women were adequately treated, consistent with other studies (16, 19, 26–27). These findings point to the need to qualify the health care network for diagnosis and management of syphilis in pregnant women, with the availability of rapid tests in the primary health care, adequate training of professionals and active search of partners for diagnosis and treatment.

In the cluster with lower incidence levels, diagnosis during prenatal care and adequate treatment of pregnant women showed better indicators. These findings emphasize that appropriate diagnosis and treatment with benzathine penicillin (1, 8) during pregnancy are effective measures in the prevention of syphilis repercussions, especially in the neonate (17, 21, 26, 27, 29). It is also worth mentioning that tests and treatment for maternal syphilis are inexpensive (30), especially when compared to the interventions in the child affected with CS (31), such as hospitalization costs and serological and imaging tests (7, 32).

Regarding the high proportion of untreated partners, similar findings were obtained on other national studies (16, 18, 26, 27, 28, 33). These findings may be a consequence of the Informative Note N. 2-SEI/2017 (11, 34), which stated that treatment of the pregnant woman's partner is no longer considered for the definition of CS. However, it is extremely important in order to avoid reinfection and poor outcomes during pregnancy (15, 18, 30, 31). Another particularity of the partner's treatment is the need for an effective communication between the pregnant women and their respective partners about the infection, which involves the fear of retaliation, fear of breaking the relationship and fear of being blamed for contracting the disease (15).

The treatment of the pregnant woman's partner was the indicator with the least favorable completion, with a high frequency of the field “ignored” (29%). Among partners that performed treatment, it was not possible to analyze whether treatment was appropriate, due to the lack of data in the information system. Incomplete fields on the notification form were common—most variables presented losses above 7%, with the exception of mother's age, in which the field “ignored” totalized 4%, and final diagnosis of the neonate, the only field filled out properly, although not the only mandatory variable analyzed in this study, such as maternal treatment and diagnosis, prenatal care, race, and maternal education (35). Therefore, training health professionals to fill in individual notification forms is urgent, as well as the awareness of the importance of notification, so that accurate data can support the planning of effective prevention actions in public health.

Despite the described scenario, Brazil has been adopting measures in order to reduce the transmission of CS, such as purchasing and distribution centralization of supplies for diagnosis and treatment, production of strategic information tools for public health managers and investments in syphilis (11). Even though Brazil has a solid system for notification and data gathering on CS (31), it is essential to adopt strategies in order to achieve accurate information, ranging from the availability of health supplies for diagnosis and treatment of syphilis to the adequate completion of notification forms and inclusion of information on the database. Considering that CS is a preventable disease (7, 18, 32), an appropriate prenatal care is crucial, capable of reducing incidence of the disease to less than 0.5/1,000 live births (20). Thus, the precariousness of the assistance remains as a main aspect responsible for maintaining the high incidence of CS and distancing from the WHO target (30).

Limitations of the study included the use of secondary data, the inadequate completion of the notification forms and the non-specificity of the variables “prenatal care” (for not informing the number of consultations), “appropriate maternal treatment” (for not addressing the diagnostic phase of maternal syphilis and the treatment scheme used) and “partnership treatment” (for not specifying whether treatment was adequate). It is also worth mentioning that the notification of the municipality of diagnosis instead of the mother's residence, as well as the existence of polarizing municipalities, which concentrated the majority of cases, also consolidated as important limitations. Nevertheless, the growth on coefficients in the analyzed territory is notorious. The study findings have the potential to guide several public policies—improvement in data filling and appropriation, training of health and administrative professionals, and prioritization of areas with greater need for health care intervention, thus reducing incidence of CS and its impacts on the lives of children and their families.

## Data Availability

The original contributions presented in the study are included in the article/Supplementary Material, further inquiries can be directed to the corresponding author.

## References

[1] BRAZIL, Ministry of Health. Clinical protocol and therapeutic guidelines for the care of people with sexually transmitted infections (STIs). Secretary of Health Surveillance, Department of Diseases, Chronic Conditions and Sexually Transmitted Infections. Brasilia: Ministry of Health (2020). p. 1–248.

[2] Padovani C Oliveira RR Pelloso SM. Syphilis in pregnancy: association of maternal and perinatal characteristics in a southern region of Brazil. Rev Lat Am Enfermagem. (2018) 26:e319. 10.1590/1518-8345.2305.3019 PMC6091379 30110097

[3] Klausner JD. The sound of silence: missing the opportunity to save lives at birth. Bull World Health Organ. (2013) 91(9):158–158A. 10.2471/BLT.13.118604 23476083 PMC3590629

[4] Korenromp EL Rowley J Alonso M Mello MB Wijesooriya NS Mahiané SG Global burden of maternal and congenital syphilis and associated adverse birth outcomes—estimates for 2016 and progress since 2012. PLoS One. (2019) 14(7):e0211720. 10.1371/journal.pone.0211720 30811406 PMC6392238

[5] BRAZIL. Ordinance N.542 of December 22, 1986. Federal Official Gazette (Section I), 19827. (1986). Available at: https://pesquisa.bvsalud.org/ses/resource/pt/crt-3619 (Accessed October 31, 2019).

[6] Pan American Health Organization. Regional initiative for the elimination of mother-to-child transmission of HIV and congenital syphilis in Latin America and the Caribbean: concept paper. Montevideo: CLAP/SMR. (2009). 32 p. Available at: https://iris.paho.org/handle/10665.2/49406 (Accessed April 4, 2020). (Organización Panamericana de la Salud. Iniciativa regional para la eliminación de la transmisión maternoinfantil de VIH y de la sífilis congénita en América Latina y el Caribe: documento conceptual. Montevideo: CLAP/SMR. Sep 2009.32 p.).

[7] BRAZIL, Health Ministry. Vertical transmission investigation protocol. Health Ministry (2014). p. 1–84.

[8] Pan American Health Organization. ETMI PLUS Framework for the elimination of PLUS mother-to-child transmission of HIV, syphilis, hepatitis and Chagas disease. Washington, DC: PAHO (2017). p. 1–30. Available at: https://www.paho.org/es/documentos/etmi-plus-marco-para-eliminacion-transmision-maternoinfantil-vih-sifilis-hepatitis (Accessed April 5, 2020). (Organización Panamericana de la Salud. ETMI PLUS Marco para la eliminación de PLUS la transmisión maternoinfantil del VIH, la sífilis, la hepatitis y y la enfermedad de Chagas. Washington, DC: OPS; 2017. 1-30 p.).

[9] World Health Organization. Global health sector strategy on sexually transmitted infections 2016–2021: toward ending STIs. Geneva (2016). p. 1–64. Available at: https://apps.who.int/iris/bitstream/handle/10665/246296/WHO-RHR-16.09-eng.pdf?sequence=1 (Accessed April 5, 2020).

[10] Pan American Health Organization. New generations free of HIV, syphilis, hepatitis B and Chagas disease in the Americas 2018: EMTCT Plus. Washington, DC: PAHO (2018). p. 4–52.

[11] BRAZIL, Ministry of Health. Syphilis epidemiological bulletin 2019. Health surveillance secretariat department of diseases, chronic conditions and sexually transmitted infections – DCCI. Brasilia: Ministry of Health (2019). 43 p.

[12] BRAZIL, Ministry of Health, Secretary of Health Surveillance. Health surveillance guide. 3rd ed. Brasília: Ministry of Health (2019). 740 p.

[13] Almeida E. Estimating spatial dependence. In: Applied spatial econometrics. 1st ed. Campinas, São Paulo: Editora Alínea (2012). p. 1–498. (Almeida E. Estimando a dependência espacial. In: Econometria espacial aplicada. 1a Edição. Editora Alínea. Campinas, São Paulo (2012). p. 1–498).

[14] Morgenstern H. Ecologic studies. In: Rothman KJ Greenland S, editors. Modern epidemiology. 2nd ed. Philadelphia: Lippincott-Raven Publishers (1998). p. 459–480.

[15] Figueiredo DCMM Figueiredo AM Souza TKB Tavares G Vianna RP. Relation between provision of diagnosis and treatment of syphilis in primary care on the incidence of gestational and congenital syphilis. Cad Saúde Pública. (2020) 36(3):e00074519. 10.1590/0102-311X00074519 32215510

[16] Heringer ALDS Kawa H Fonseca SC Brignol SMS Zarpellon LA Reis AC. Inequalities in congenital syphilis trends in the city of Niterói, Brazil, 2007–2016. Rev Panam Salud Publica. (2020) 44:e3. 10.26633/RPSP.2020.8 32038724 PMC7001125

[17] Domingues RM Leal Mdo C. Incidence of congenital syphilis and factors associated with vertical transmission: data from the Birth in Brazil study. Cad Saude Publica. (2016) 32(6):1–12. 10.1590/0102-311X00082415 27333146

[18] de Almeida MFG Pereira SM. Epidemiological characterization of congenital syphilis in the city of Salvador, Bahia. STD – J Bras Dis. Sex Transm. (2007) 19(3–4):144–56.

[19] Reis GJD Barcellos C Pedroso MM Xavier DR. Intraurban differentials of congenital syphilis: predictive analysis by neighborhoods in the city of Rio de Janeiro, Brazil. Cad Saúde Pública. (2018) 34(9):e00105517. 10.1590/0102-311x00105517 30208175

[20] Araújo CLD Shimizu HE Sousa AIAD Hamann EM. Incidence of congenital syphilis in Brazil and its relationship with the Family Health Strategy. Rev Saúde Pública. (2012) 46:479–486. 10.1590/S0034-89102012000300010 22635036

[21] Cavalcante PADM Pereira RBDL Castro JGD. Sífilis gestacional e congênita em Palmas, Tocantins, 2007–2014. Epidemiol Serv Saude. (2017) 26:255–264. 10.5123/S1679-49742017000200003 28492767

[22] BRAZIL, Ministry of Health. Syphilis Epidemiological Bulletin 2017. Vol. 48, n.36, Secretariat of Health Surveillance, Department of Surveillance, Prevention and Control of STIs, HIV/AIDS and Viral Hepatitis – DIAVH/SVS/MS. Brasilia: Ministry of Health. (2017). p. 1–44. Available at: http://www.aids.gov.br/pt-br/pub/2017/boletim-epidemiologico-de-sifilis-2017 (Accessed April 15, 2020).

[23] Ministry of Health. Joint Information Note n°109/105/GAB/SVS/MS. Brasilia: Ministry of Health (2015). 3 p. Available at: http://antigo.aids.gov.br/pt-br/legislacao/nota-informativa-conjunta-no-109105gabsvsms-gabsctiems (Accessed May 4, 2021).

[24] State of Bahia. About Cedap. State of Bahia. Secretary of Health (2020). Available at: http://www.saude.ba.gov.br/cedap (Accessed April 15, 2020).

[25] Nunes PS Guimarães RA Rosado LEP Marinho TA Aquino ÉC Turchi MD. Temporal trend and spatial distribution of syphilis in pregnancy and congenital syphilis in Goiás, Brazil, 2007–2017: an ecological study. Epidemiol Serv Saude. (2021) 30(1):e2019371. 10.1590/S1679-49742021000100002 33503212

[26] Marinho de Souza J Giuffrida R Ramos APM Morceli G Coelho CH Pimenta Rodrigues MV. Mother-to-child transmission and gestational syphilis: spatial-temporal epidemiology and demographics in a Brazilian region. PLoS Negl Trop Dis. (2019) 13(2):e0007122. 10.1371/journal.pntd.0007122 30789909 PMC6383870

[27] Vescovi JS Schuelter-Trevisol F. Increased incidence of congenital syphilis in the state of Santa Catarina from 2007 to 2017: analysis of the temporal trend. Rev Paul Pediatr. (2020) 38:e2018390. 10.1590/1984-0462/2020/38/2018390 32667471 PMC7357596

[28] Oliveira LR Costa Mda C Barreto FR Pereira SM Dourado I Teixeira MG. Evaluation of preventative and control measures for congenital syphilis in the State of Mato Grosso. Rev Soc Bras Med Trop. (2014) 47(3):334–40. 10.1590/0037-8682-0030-2014 25075485

[29] Nonato SM Melo APS Guimaraes MDC. Syphilis during pregnancy and factors associated with congenital syphilis in Belo Horizonte-MG, 2010–2013. Epidemiol Serv Saude. (2015) 24(4):681–94. 10.5123/S1679-49742015000400010

[30] Saloojee H Velaphi S Goga Y Afadapa N Steen R Lincetto O. The prevention and management of congenital syphilis: an overview and recommendations. Bull World Health Organ. (2004) 82(6):424–30. 15356934 PMC2622853

[31] Newman L Kamb M Hawkes S Gomez G Say L Seuc A Global estimates of syphilis in pregnancy and associated adverse outcomes: analysis of multinational antenatal surveillance data. PLoS Med. (2013) 10(2):e1001396. 10.1371/journal.pmed.1001396 23468598 PMC3582608

[32] Pan American Health Organization. Elimination of Neglected Diseases and Other Poverty-Related Infections. Washington (2009). p. 1–12 (Resolution CD49.R19). Available at: https://iris.paho.org/handle/10665.2/399 (Accessed April 15, 2020).

[33] Serafim AS Moretti GP Serafim GS Niero CV Rosa MI Pires MM Incidence of congenital syphilis in the South Region of Brazil. Rev Soc Bras Med Trop. (2014) 47(2):170–8. 10.1590/0037-8682-0045-2014 24861290

[34] BRAZIL, Ministry of Health, Department of Surveillance, Prevention and Control of STIs, HIV/AIDS and Viral Hepatitis. Information Note No 2 -SEI/2017: Changes the case definition criteria for reporting acquired syphilis, syphilis in pregnant women and congenital syphilis. Changes the case definition criteria for notification of acquired syphilis, syphilis in pregnant women and congenital syphilis. Brasília: Ministry of Health (2017). p. 1–5. Available at: http://portalsinan.saude.gov.br/images/documentos/Agravos/Sifilis-Ges/Nota_Informativa_Sifilis.pdf (Accessed April 4, 2020).

[35] BRAZIL, Ministry of Health, Secretary of Health Surveillance. Notifiable diseases information system - SINAN net data dictionary. Grievance: Congenital Syphilis, Ministry of Health (2012). p. 1–20. Available at: http://portalsinan.saude.gov.br/images/documentos/Agravos/Sifilis-Con/DIC_DADOS_Sifilis_Congenita_v5.pdf (Accessed May 10, 2021).

